# Supplementary material for: The sodium/glucose cotransporter 2 inhibitor empagliflozin is a pharmacological chaperone of cardiac Nav1.5 channels
Source: Am J Physiol Heart Circ Physiol. Author manuscript; Available in PMC 2025 Sep 15. (PMC7618122; doi:10.1152/ajpheart.00363.2025)
Supplement: Supplemental Material — Supplemental Figs. S1–S6 and Table S1 are available via FigShare: https://doi.org/10.6084/m9.figshare.29560217.v1. [file EMS207943-supplement-Supplemental_Material.pdf]

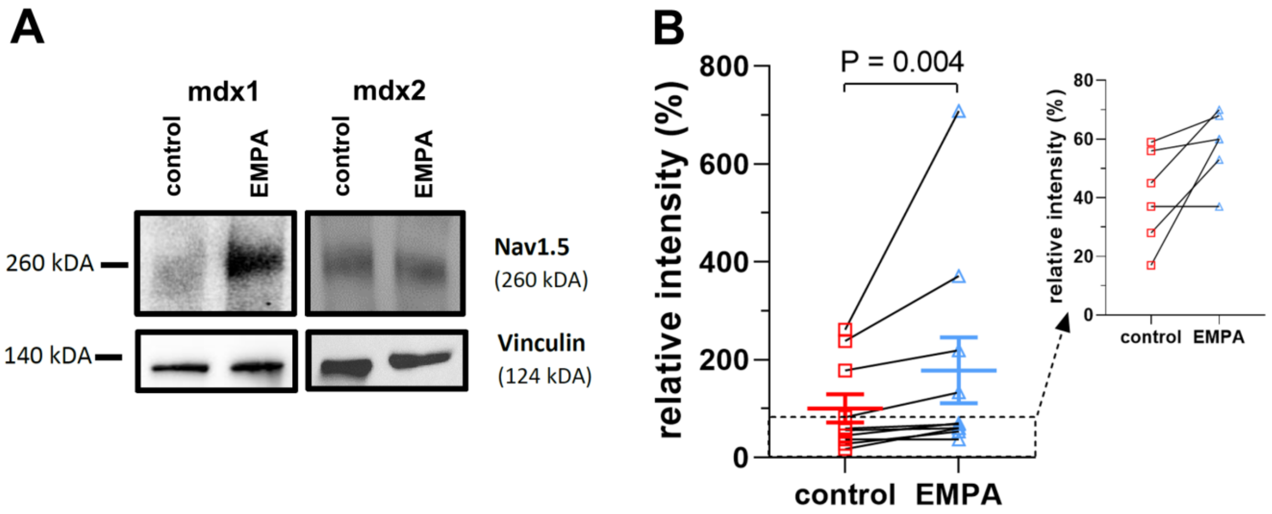

Supplemental Figure 1

Chronic exposure to EMPA increases $Na_v1.5$ protein levels in isolated mdx cardiomyocytes. **A**, Representative western blots of membrane fractions of the protein lysate from ventricular cardiomyocytes originating from two mdx hearts. Isolated cardiomyocytes had been incubated for 24 h with 1 µM EMPA, or under control conditions. **B**, Relative band intensities of $Na_v1.5$ for control and EMPA-treated cardiomyocytes from 10 mdx hearts. $Na_v1.5$ intensities were normalized to the respective band intensities of vinculin, and then to the mean relative intensity for the control group. Means ± SE are displayed. Each dot represents the cells isolated from one heart. Lines connect control and EMPA-treated cells originating from the same heart. A Wilcoxon matched-pairs signed rank test revealed a significant difference between the two groups. The inset provides a clearer comparison of the relative intensities up to 80 %.

Methods: western blotting

Isolated mdx ventricular cardiomyocytes were incubated for a 24 h period with EMPA or solvent. For protein isolation, first, the cardiomyocytes were washed with ice-cold PBS. Then, they were lysed using a buffer containing 50 mM Tris, 150 mM NaCl, 10 mM N-ethyl maleimide, 1 mM EGTA, 1 % Triton X-100, 100 µM PMSF, 100 µM leupeptin and 100 µM aprotinin (adjusted to pH 7.5 with HCl) (modified from Albesa et al., DOI: 10.1093/cvr/cvq326) to extract membrane proteins. The cell lysate was sonicated for 2 min and then centrifuged for 45 min at 50,000 *g* at 4 °C. The supernatant was collected, and the protein concentration was measured using Pierce™ BCA Protein Assay Reagent A (Thermo Fisher Scientific, catalog number 23222) and $CuSO_4$. Sample buffer (60 mM Tris, pH 6.8, 14.4 mM β-mercaptoethanol, 25 % glycerine, 2 % SDS and 0.1 % bromophenol blue) was diluted 1:4 with the protein lysate, and the mixture was heated to 45 °C for 10 min. For protein size separation, a pre-cast 1-mm-thick Mini-PROTEAN® TGX™ 4-15 %, 50 µl, 10-well (Bio-Rad, catalogue number 4561084) was

used. The electrophoresis gel was loaded with 40 µg protein per sample. The gel was first run for 10 min with 70 V and then for 55 min with 200 V (modified from Boixel et al., DOI: 10.1152/ajpheart.01060.2005). Separated proteins were blotted onto a nitrocellulose membrane using wet transfer with the Mini Trans-Blot Electrophoretic Transfer Cell (Bio-Rad). It was run at constant 100 V for 3 h whilst keeping the chamber cool in an ice bath (modified from Boixel et al., DOI: 10.1152/ajpheart.01060.2005). The transfer buffer contained 25 mM Tris, 192 mM glycine and 20 % methanol. Ponceau S and coomassie blue stainings were performed to confirm proper protein transfer. Membranes were blocked with 5 % skim milk in Tris-buffered saline with 0.1 % Tween 20 and 1 mM EDTA (py-TBST). The antibody used for $Na_v1.5$ detection was an anti-$Na_v1.5$ rabbit monoclonal antibody (Cell Signaling Technology, catalogue number 14421) diluted 1:1000 in py-TBST without BSA. Vinculin was used as a loading control and detected with an anti-vinculin rabbit monoclonal antibody (Cell Signaling Technology, catalogue number 13901) diluted 1:1000 in py-TBST with 5 % BSA, according to the manufacturer's instructions. The membrane was incubated with the primary antibody for 24 h at 4 °C with gentle shaking. After 24 h, the membrane was washed three times for 5 min each with py-TBST and then incubated with the secondary antibody (horseradish peroxidase-conjugated anti-rabbit secondary antibody, Cell Signaling Technology, catalogue number 7074S) for 1 h. After three washing steps (5 min each) with py-TBST and one washing step with TBS (10 min), the protein was detected by ECL (Pierce™ ECL Plus Western Blotting Substrate, Thermo Fisher Scientific, catalogue number 32132) and the ChemiDoc Imaging System (Bio-Rad). Analysis was performed with Image Studio (LICOR).

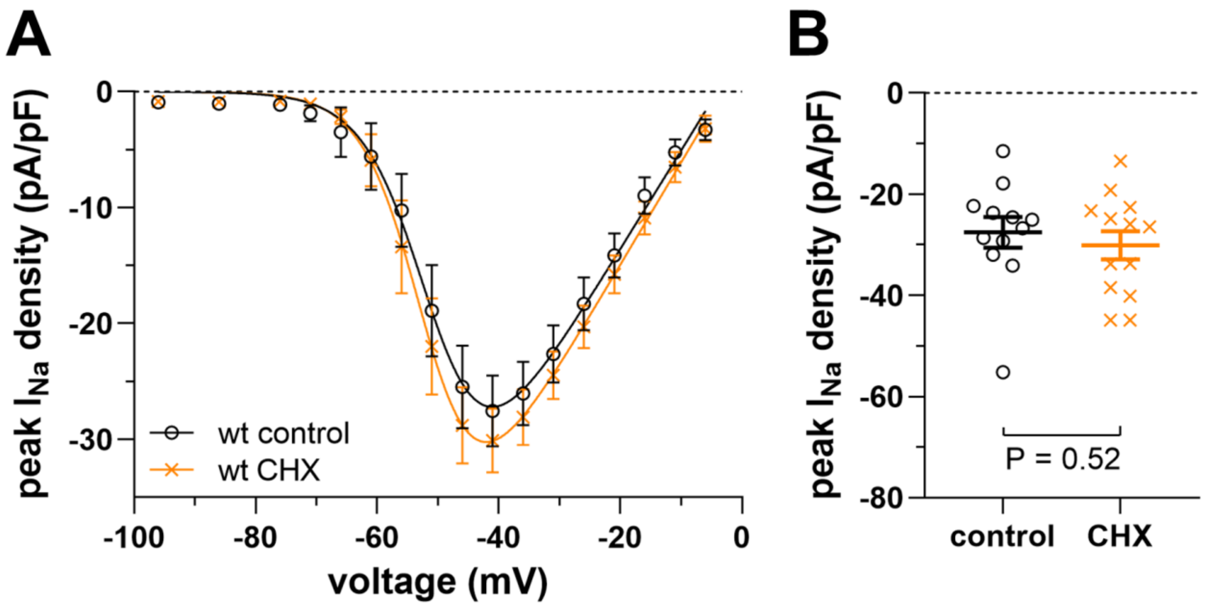

Supplemental Figure 2

Cycloheximide (CHX) does not affect peak I_{Na} of ventricular cardiomyocytes isolated from wild-type (wt) mice. **A**, Peak I_{Na} density-voltage relationships from wt cardiomyocytes incubated for 4 h under control conditions (n = 12 cells), or in the presence of 50 µg/ml CHX (n = 13 cells, all cells originating from the same 3 wt hearts). **B**, Respective dot plot (at -41 mV) showing no significant difference between control and CHX-treated cells.

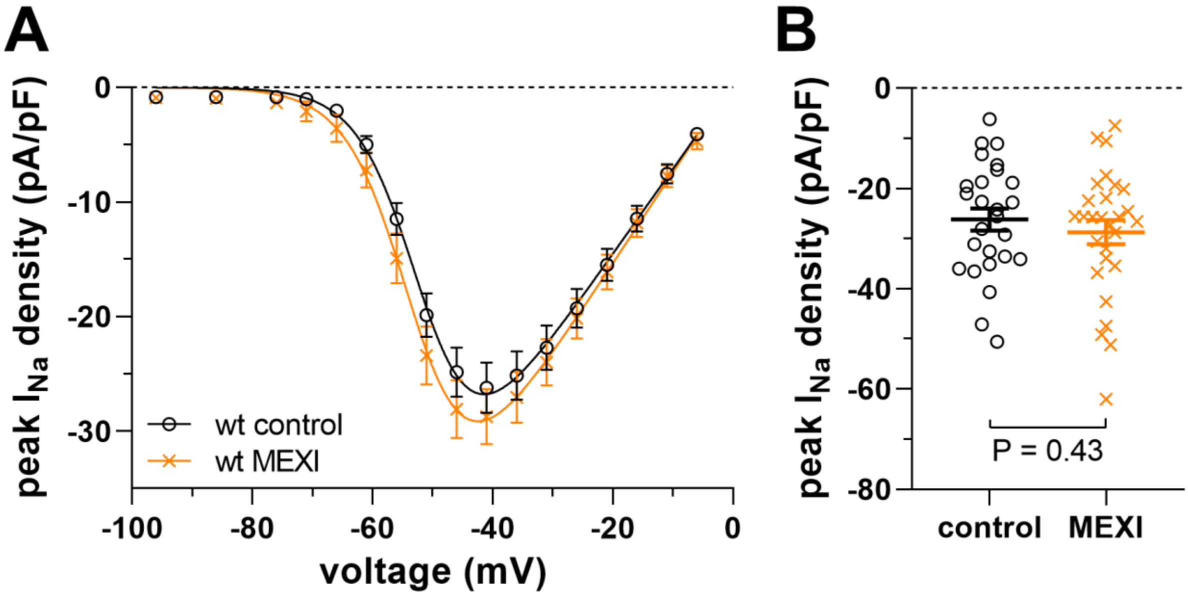

Supplemental Figure 3

Chronic mexiletine (MEXI) treatment does not increase peak I_{Na} of ventricular cardiomyocytes isolated from wild-type (wt) mice. **A**, Peak I_{Na} density-voltage relationships from wt cardiomyocytes incubated for 24 h under control conditions (n = 26 cells), or in the presence of 10 µM MEXI (n = 28 cells, all cells originating from the same 4 wt hearts). **B**, Respective dot plot (at -41 mV) showing no significant difference between control and MEXI-treated cells.

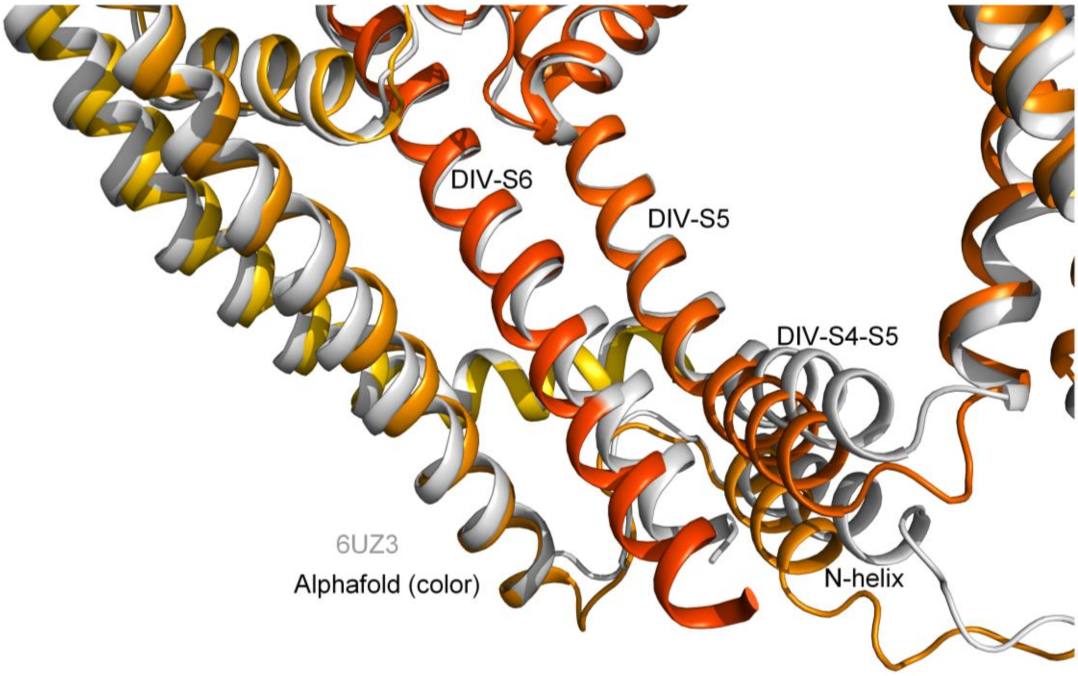

Supplemental Figure 4

Cartoon representation of DIVS6 and surrounding helices in the Alphafold model shown in color, with the 6UZ3 structure aligned with Swiss-pdb viewer (Guex and Peitsch, 1997; https://pubmed.ncbi.nlm.nih.gov/9504803/) shown in gray. Inward rotation of DIV-S6 is accompanied by movements of the DIV-S4-S5 linker (inward, downward) and the N-helix (inward, downward). Changes in the VSD were not analyzed, since they are not in the vicinity of the drug binding interface.

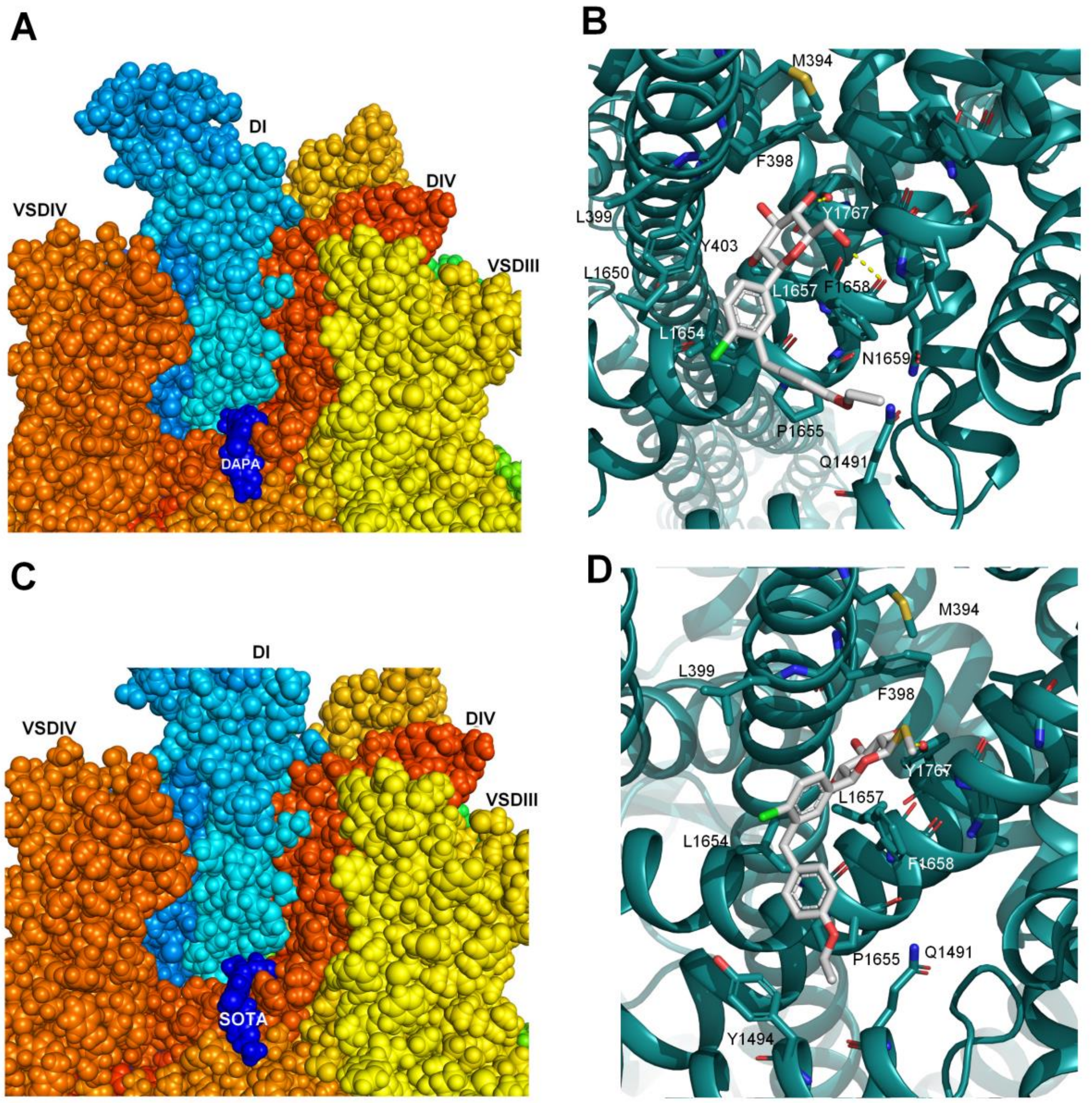

Supplemental Figure 5

Consensus binding modes of DAPA and SOTA. **A**, **C**, spheres representation of the consensus binding modes of DAPA and SOTA at the fenestration of DI-DIV shown in side view. **B**, **D**, close-up view of residues within 5 Å of the drugs, shown as sticks. Hydrogen bonds are shown as yellow dotted lines.

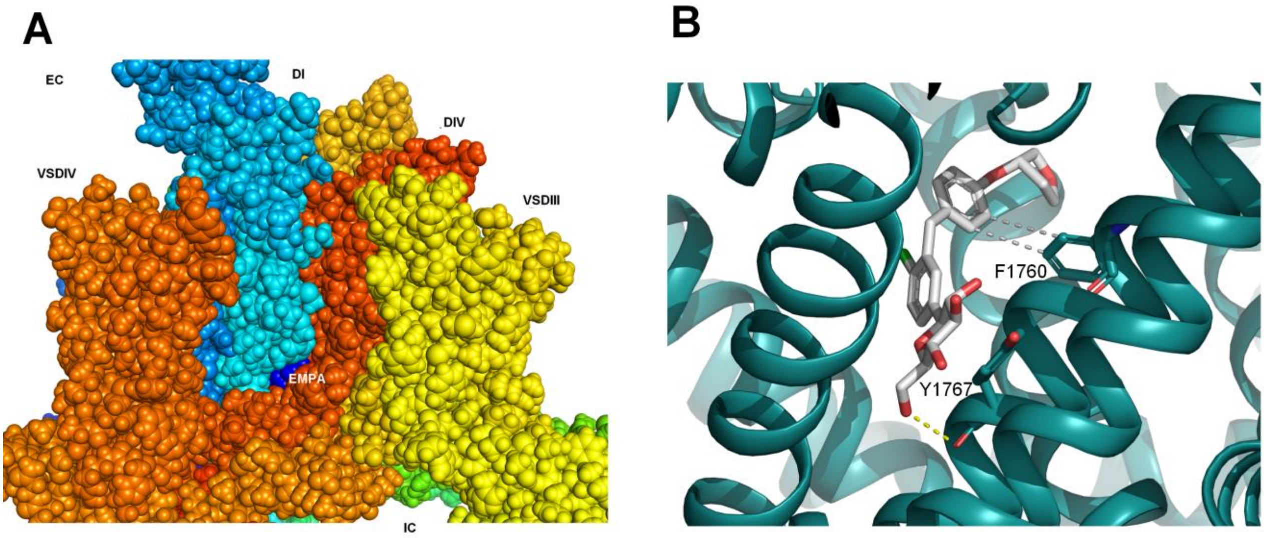

Supplemental Figure 6

Alternative binding mode of EMPA (~ 10% of poses). **A**, side view of EMPA binding pose with interactions with F1760 and Y1767 shown in spheres representation. **B**, hydrogen bonds to the backbone of the Y1767 side chain are shown as yellow dotted sticks, hydrophobic interactions between the 4-(tetrahydrofuran-3-yloxy) benzyl group of EMPA and the aromatic side chain of F1760 are indicated as gray dotted lines.

Supplemental Table 1

PDB	Quality	Too close contacts with amino acids/small molecules
**7XSU**<br>**rat Nav1.5**	No issues	-
**8T6L**<br>**rat Nav1.5**	Too close contact	ASN407, 0.42 Å clash overlap
**6UZ0**<br>**rat Nav1.5**	Too close contacts	ASN407, 0.64 Å clash overlap + within Y1767
**6UZ3**<br>**rat Nav1.5**	Too close contacts	ASN407, 0.64 Å clash overlap + within Y1767
**7FBS**<br>**rat Nav1.5**	Too close contact	(3beta,14beta,17beta,25R)-3-[4-methoxy-3-(methoxymethyl)butoxy]spirost-5-en, 0.4 Å clash overlap
**7K18**<br>**rat Nav1.5**	No issues	-
**8F6P**<br>**rat Nav1.5**	Too close contacts	VAL1765, 0.67 Å clash overlap; LEU1659, 0.59 Å clash overlap
**6LQA**<br>**human Nav1.5**	Too close contacts	Several clashes within Y1767, up to 0.85 Å
**7DTC**<br>**human Nav1.5**	Too close contact	VAL1764, 0.44 Å

Despite the fact that cryo-EM structures 7XSU and 7K18 do not show problematic geometry at the region of Y1767, these structures were not considered for docking, since they do not represent a “fully closed” activation gate, due to toxin binding to the VSD of domain IV (Jiang et al., 2021; https://pubmed.ncbi.nlm.nih.gov/33397917/).
